# Depression in Atrial Fibrillation in the General Population

**DOI:** 10.1371/journal.pone.0079109

**Published:** 2013-12-04

**Authors:** Renate B. Schnabel, Matthias Michal, Sandra Wilde, Jörg Wiltink, Philipp S. Wild, Christoph R. Sinning, Edith Lubos, Francisco M. Ojeda, Tanja Zeller, Thomas Munzel, Stefan Blankenberg, Manfred E. Beutel

**Affiliations:** 1 Department of General and Interventional Cardiology, University Heart Center, Hamburg, Germany; 2 Department of Psychosomatic Medicine and Psychotherapy, University Medical Center of the Johannes Gutenberg-University, Mainz, Germany; 3 Department of Medicine 2, University Medical Center of the Johannes Gutenberg-University, Mainz, Germany; 4 Center of Thrombosis and Hemostasis University Medical Center of the Johannes Gutenberg-University, Mainz, Germany; Pulmonary Research Institute at LungClinic Grosshansdorf, United States of America

## Abstract

**Background:**

Initial evidence suggests that depressive symptoms are more frequent in patients with atrial fibrillation. Data from the general population are limited.

**Methods and Results:**

In 10,000 individuals (mean age 56±11 years, 49.4% women) of the population-based Gutenberg Health Study we assessed depression by the Patient Health Questionnaire (PHQ-9) and a history of depression in relation to manifest atrial fibrillation (n = 309 cases). The median (25th/75th percentile) PHQ-9 score of depressive symptoms was 4 (2/6) in atrial fibrillation individuals versus 3 (2/6) individuals without atrial fibrillation, 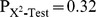. Multivariable regression analyses of the severity of depressive symptoms in relation to atrial fibrillation in cardiovascular risk factor adjusted models revealed a relation of PHQ-9 values and atrial fibrillation (odds ratio (OR) 1.04, 95% confidence interval (CI) 1.01–1.08; P = 0.023). The association was stronger for the somatic symptom dimension of depression (OR 1.08, 95% CI 1.02–1.15; P = 0.0085) than for cognitive symptoms (OR 1.05, 95% CI 0.98–1.11; P = 0.15). Results did not change markedly after additional adjustment for heart failure, partnership status or the inflammatory biomarker C-reactive protein. Both, self-reported physical health status, very good/good versus fair/bad, (OR 0.54, 95% CI 0.41–0.70; P<0.001) and mental health status (OR 0.61 (0.46–0.82); P = 0.0012) were associated with atrial fibrillation in multivariable-adjusted models.

**Conclusions:**

In a population-based sample we observed a higher burden of depressive symptoms driven by somatic symptom dimensions in individuals with atrial fibrillation. Depression was associated with a worse perception of physical or mental health status. Whether screening and treatment of depressive symptoms modulates disease progression and outcome needs to be shown.

## Introduction

Atrial fibrillation (AF) and its sequelae have become a significant public health burden and cost factor in the health care system due to an increasing prevalence in aging populations [1]. Although individuals with AF are at high risk of incident stroke and heart failure, an increased risk of death remains after accounting for these serious complications and other cardiovascular comorbidities. Numerous studies have found that depression predicts prognosis in cardiac conditions such as stable coronary artery disease, myocardial infarction, and heart failure [2]. Evidence suggests that depressive symptoms in AF are related to the recurrence of AF episodes [3] and the occurrence of complications of AF such as heart failure and death in the clinical setting [4]. From other cardiovascular diseases we know that psychosocial distress may influence hemodynamics, vascular function, autonomic tone, inflammatory activity, and hemostasis [5], [6], [7], all of which play a role in the pathogenesis and complications of AF.

Initial smaller investigations in AF indicate that the disease is frequently accompanied by depressive symptoms which may impact physical activity and quality of life in AF patients [8], [3], [9]. However, overall there is a critical lack of knowledge regarding the type and extent of psychological distress and its consequences in patients diagnosed with AF [10]. Data in individuals with AF in the general population are rare. We hypothesized that in ambulatory individuals from the general population depression is more frequent in AF independent of age, sex and cardiovascular comorbidities.

## Methods

### Ethics statement

Prior to enrolment participants signed written, informed consent. The study has been approved by the local Ethics Committee (Landesaerztekammer Rheinland-Pfalz, 837.020.07).

### Study participants

The Gutenberg Health Study constitutes a cohort of a randomly selected population-based sample of the region of Mainz/Mainz-Bingen aged 35 to 74 years with a proportion of 49% women. It was incepted in 2007 at the Department of Medicine 2, University Medical Center Mainz. During the baseline clinic visit comprehensive information on cardiovascular risk factors is collected by anthropometric measures and standardized computer-assisted interview. Classical cardiovascular risk factors were defined as follows: Smoking status comprised the categories non-smokers (never smokers and former smokers) and smokers. Diabetes mellitus was diagnosed when individuals reported a physician diagnosis of diabetes and/or a fasting blood glucose concentration of ≥126 mg/dL (minimum 8-hour fast) or a blood glucose level of ≥200 mg/dL at any time was measured on site. Dyslipidemia was defined based on a physician's diagnosis of dyslipidemia and/or a low-density-lipoprotein/high-density-lipoprotein cholesterol ratio of >3.5. The definition of hypertension comprised anti-hypertensive drug treatment and/or a mean systolic blood pressure of ≥140 mmHg and/or a mean diastolic blood pressure of ≥90 mmHg.

Myocardial infarction was assessed by self-report. Heart failure was defined by self-reported treatment of heart failure within the last 12 months.

Depression was assessed by the Patient Health Questionnaire (PHQ-9), which quantifies the frequency of being bothered by each of the 9 diagnostic criteria of Major Depression over the past 2 weeks. Responses are summed to create a score between 0 and 27 points. A PHQ-9 sum score of ≥10 was used for the definition of caseness for depression [11]. The somatic and cognitive dimensions of depression were defined according to prior studies [12], [13], [14], [15], [16]. Four PHQ-9 items related to problems with sleep, fatigability, appetite, and psychomotor agitation/retardation were classified as somatic depressive symptoms, whereas 5 items, related to lack of interest, depressed mood, negative feelings about self, concentration problems and suicidal ideation, were classified as cognitive depressive symptoms. During the computer-assisted personal interview participants were asked whether they had ever received the definite diagnosis of any depressive disorder by a physician (medical history of lifetime diagnosis of any depressive disorder).

Socioeconomic status was evaluated using Lampert's and Kroll's Score of socioeconomic status that ranges from 3 to 27 with 3 indicating the lowest socioeconomic status.

The diagnosis of AF was made based on a history of AF reported by the participant and/or the electrocardiographic documentation (GE Cardiosoft®) of AF or atrial flutter [17]. At least two physicians with cardiology training and experience in electrocardiogram (ECG) reading had to agree on the diagnosis.

C-reactive protein (CRP) was measured from fasting blood samples by a standardized method through particle enhanced turbidimetric immunoassay (Dimension® Chemistry System, Dade Behring).

All authors have read and approved the manuscript as written.

### Statistical methods

Data were analyzed for the first 10,000 consecutive GHS individuals. We did not require complete case analysis so that in some analyses the total number of individuals may be smaller than the total sample size. Demographics and prevalence of risk factors for the study sample weighted for the population of the region Mainz/Mainz-Bingen are also provided in the supplement. Since depression may depend on awareness of the disease AF, we also present data separately for individuals with a prior diagnosis of AF and participants with a first diagnosis of AF on the study ECG. In logistic regression models, depression and its dimensions were related to atrial fibrillation. Models were adjusted for age and sex as well as for age, sex and atrial fibrillation risk factors body mass index, systolic blood pressure, antihypertensive medication, diabetes, current smoking, dyslipidemia and a family history of myocardial infarction. Model R^2^ values were calculated. Additional models were calculated adjusting for heart failure, partnership status or CRP. We tested for interactions of depressive symptoms in association with AF by age and sex.

To understand whether depression and its symptom dimensions are mediated by other psychosocial conditions, cardiac function or inflammatory activity, we describe depressive symptoms according to partnership, manifest heart failure, and median CRP concentrations. Due to expectedly lower numbers in the subgroup of individuals with AF we performed analyses for very good/good versus fair/bad self-rated physical and mental health status.

In secondary analyses we also performed multivariable regression analyses of depressive symptom dimensions in relation to self-rated physical and mental health status for individuals with AF.

We assumed a threshold of *P*<0.05 for statistical significance. As this is an explorative study no adjustments for multiple testing have been performed. *P* values are given for descriptive reasons.

Statistical calculations were performed using R software, Version 2.14.0 (R Development Core Team, 2009). R: A language and environment for statistical computing. R Foundation for Statistical Computing, Vienna, Austria. ISBN 3-900051-07-0, http://www.r-project.org/)

## Results

The study sample by AF status is described in **Table 1**. Individuals with AF were older (mean±standard deviation (SD)) 64.8±8.2 versus 55.2±10.8 years in individuals without AF, less than one third was female. Except for smoking cardiovascular risk factors were more frequent in AF. CRP concentrations were higher 3.6±4.1 mg/L than in individuals without a diagnosis of AF 2.9±5.5 mg/L. A history of depression was documented in 16.2% of AF individuals. The median (25^th^/75^th^ percentile) PHQ-9 value was 4 (2/6) compared to 3 (2/6) participants without AF. Of individuals with AF 34.9% reported impaired physical health, 21.4% reduced mental health compared to 20.3% and 17.2%, respectively, in the rest of the sample. Data weighted for the total population were comparable as shown in **Table S1**. Variable distribution was similar in individuals with a history of AF and newly diagnosed AF (**Table S2**).

**Table 1:** Characteristics of the sample by AF status.

Variable	No Atrial Fibrillation N = 9680	Atrial Fibrillation N = 309
Age, years	55.2±10.8	64.8±8.2
Female gender, N (%)	4846 (50.1)	93 (30.1)
Partnership, N (%)	7898 (81.6)	253 (81.9)
Socioeconomic status	12.8±4.5	11.8±4.7
Body mass index, kg/m^2^	26.62 (23.96/30.02)	28.39 (25.95/32.16)
Systolic blood pressure (mmHg)	132.3±17.6	132.8±17.3
Smoking, N (%)	1870 (19.4)	41 (13.4)
Diabetes, N (%)	711 (7.4)	47 (15.2)
Hypertension, N (%)	4962 (51.3)	223 (72.2)
Family history of myocardial infarction, N (%)	1616 (16.7)	56 (18.1)
History of heart failure, N (%)	99 (1.0)	37 (12.0)
History of myocardial infarction, N (%)	260 (2.7)	42 (13.9)
CRP, mg/L	2.9±5.5	3.6±4.1
Depression		
History of depression, N (%)	1471 (15.4)	49 (16.2)
Severity of depression by PHQ-9 (range 0–27)	3 (2/6)	4 (2/6)
Caseness of depression (PHQ-9≥10)	705 (7.4)	18 (6.0)
Somatic depression (0–12)	2 (1/3)	2 (1/4)
Cognitive depression (0–15)	1 (0/2)	1 (0/2)
Mental health status		
Very good mental health status, N (%)	1808 (18.7)	44 (14.2)
Good mental health status, N (%)	6198 (64.1)	199 (64.4)
Fair mental health status, N (%)	1414 (14.6)	58 (18.8)
Poor mental health status, N (%)	251 (2.6)	8 (2.6)
Physical health status		
Very good physical health status, N (%)	1343 (13.9)	21 (6.8)
Good physical health status, N (%)	6361 (65.8)	180 (58.3)
Fair physical health status, N (%)	1686 (17.4)	86 (27.8)
Poor physical health status, N (%)	280 (2.9)	22 (7.1)

Provided are mean and standard deviation for continuous variables, or median (25^th^/75^th^ percentile) for variables with a skewed distribution. Number and percent are shown for categorical variables.

CRP stands for C-reactive protein and PHQ stands for Patient Health Questionnaire.

Multivariable regression analyses of depressive symptom dimensions in relation to AF in cardiovascular risk factor adjusted models (**Table 2**) showed an association of the degree of depressive symptoms (PHQ-9 sum score, range 0–27) and AF (odds ratio (OR) 1.04, 95% confidence interval (CI) 1.01–1.08; P = 0.023). The association was more pronounced for the somatic symptom dimension of depression, OR 1.08, 95% CI 1.02–1.15; P = 0.0085 than for cognitive depressive symptoms, OR 1.05, 95% CI 0.98–1.11; P = 0.15.

**Table 2:** Multivariable logistic regression of depressive symptom dimensions in relation to AF.

Variable	Model R^2^	Odds Ratio	*P* Value
History of depression	0.12	1.41 (1.02–1.95)	0.035
	0.16	1.21 (0.87–1.68)	0.26
Severity of depression (PHQ-9, range 0–27)	0.13	1.06 (1.03–1.10)	0.00027
	0.16	1.04 (1.01–1.08)	0.023
Caseness of depression (PHQ-9≥10)	0.12	1.17 (0.72–1.92)	0.53
	0.16	0.92 (0.55–1.54)	0.76
Somatic depression (0–12)	0.13	1.13 (1.06–1.19)	<.0001
	0.16	1.08 (1.02–1.15)	0.0085
Cognitive depression (0–15)	0.13	1.08 (1.02–1.14)	0.013
	0.16	1.05 (0.98–1.11)	0.15
Physical health status (very good/good vs. fair/poor)	0.14	0.47 (0.36–0.60)	<0.0001
	0.16	0.54 (0.41–0.70)	<0.0001
Mental health status (very good/good vs. fair/poor)	0.13	0.57 (0.43–0.76)	0.00012
	0.16	0.61 (0.46–0.82)	0.0012

Odds ratios are across categories or for the dichotomous variable as indicated. Atrial fibrillation was used as dependent variable. Multivariable-adjusted models included age, sex (upper row) and age, sex, body mass index, systolic blood pressure, antihypertensive medication, diabetes, current smoking and a family history of myocardial infarction, dyslipidemia (lower row) and respective model R^2^ values.

Worse self-reported physical health status was also associated with AF, OR 0.54, 95% CI 0.41–0.70; P<0.0001; as well as worse mental health status (OR 0.61 (0.46–0.82); P = 0.0012. Model R^2^ values were 0.16 for both. We did not identify statistically significant interactions of association between depressive symptom dimensions and AF.

Direction and magnitude of association results were similar when we restricted analyses to individuals with known AF (**Table S3**). Results were not markedly changed when multivariable-adjustment also incorporated heart failure, partnership status or CRP concentrations (data not shown).

In **Table 3** **,** **4** **and** **5** variables of depressive symptoms, health status, and socioeconomic status are demonstrated by a diagnosis of heart failure, partnership status and median CRP concentrations. None of the somatic and cognitive depressive symptoms appeared to be different in the subgroups. Self-reported very good and good mental health status reached borderline significance with lower frequency in AF individuals with heart failure (64.9% versus 80.5% in individuals without heart failure; 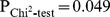). Very good and good physical health were also seen less frequently in individuals with AF (45.9% versus 67.6% in individuals without AF; 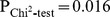) (**Table 3**). Socioeconomic status was higher in individuals with a regular partnership and lower CRP concentrations, P<0.001 (**Table 4** **and** **5**).

**Table 3:** Depressive symptom dimensions in individuals with AF by manifest heart failure.

Variable	No Heart Failure N = 272	Heart Failure N = 37	*P* Value
History of depression, N (%)	40 (15.0)	9 (25.7)	0.17
Severity of depression (PHQ-9)	3 (2/6)	4 (1/6)	0.40
Caseness of depression (PHQ-9≥10)	13 (4.9)	5 (14.7)	0.058
Somatic depression (0–12)	2 (1/3)	3 (1/5)	0.16
Cognitive depression (0–15)	1 (0/2)	1 (0/2)	0.82
Very good/good mental health status, N (%)	219 (80.5)	24 (64.9)	0.049
Very good/good physical health status, N (%)	184 (67.6)	17 (45.9)	0.016
Partnership, N (%)	224 (82.4)	29 (78.4)	0.72
Socioeconomic status	11.9±4.8	11.1±4.0	0.26

Provided are the numbers and percent of individuals or mean±standard deviation or median (25^th^/75^th^ percentile) for skewed variables. *P* values for categorical variables are according to Chi^2^-test, for continuous variables according toT-test or Mann-Whitney-U test.

**Table 4:** Depressive symptom dimensions in individuals with AF by partnership status.

Variable	PartnershipN = 253	No PartnershipN = 56	*P* Value
History of depression, N (%)	35 (14.0)	14 (26.9)	0.036
Severity of depression (PHQ-9)	3.5 (2/5)	4 (2/8)	0.11
Caseness of depression (PHQ-9≥10)	12 (4.8)	6 (11.8)	0.11
Somatic depression (0–12)	2 (1/3)	3 (1/4)	0.068
Cognitive depression (0–15)	1 (0/2)	1 (0/3)	0.24
Very good/good mental health status, N (%)	205 (81.0)	38 (67.9)	0.046
Very good/good physical health status, N (%)	170 (67.2)	31 (55.4)	0.13
Socioeconomic status	12.3±4.6	9.5±4.2	<0.001

Provided are the numbers and percent of individuals or mean±standard deviation or median (25^th^/75^th^ percentile) for skewed variables. *P* values for categorical variables are according to Chi^2^-test, for continuous variables according toT-test or Mann-Whitney-U test.

**Table 5:** Depressive symptom dimensions in individuals with AF by inflammatory activity.

Variable	CRP<Median N = 111	CRP≥Median N = 198	*P* Value
History of depression, N (%)	19 (17.6)	30 (15.5)	0.75
Severity of depression (PHQ-9)	4 (2/6)	4 (2/6)	0.89
Caseness of depression (PHQ-9≥10)	8 (7.4)	10 (5.2)	0.60
Somatic depression (0–12)	2 (1/4)	2 (1/4)	0.56
Cognitive depression (0–15)	1 (0/2)	1 (0/2)	0.98
Very good/good mental health status, N (%)	87 (78.4)	156 (78.8)	0.95
Very good/good physical health status, N (%)	76 (68.5)	125 (63.1)	0.41
Partnership, N (%)	93 (83.8)	160 (80.8)	0.62
Socioeconomic status	13.8±4.5	10.7±4.4	<0.001

Provided are the numbers and percent of individuals or mean±standard deviation or median (25^th^/75^th^ percentile) for skewed variables. The median CRP value was 1.6 mg/L. *P* values for categorical variables are according to Chi^2^-test, for continuous variables according to T-test or Mann-Whitney-U test.

In regression analyses, somatic as well as cognitive depressive symptoms were inversely related to self-reported physical and mental health status in individuals with AF in multivariable-adjusted models for a history of depression, severity of depressive symptoms assessed by PHQ-9 as well as for caseness of depression (**Table S4**).

## Discussion

In our large population-based sample, we could demonstrate a slightly higher burden of depressive symptoms in individuals with AF driven by somatic symptom dimensions independent of age, sex and classical cardiovascular risk factors. Prevalent heart failure, partnership status, or low-grade inflammation did not seem to account for depressive symptom dimensions to a relevant degree. Both, self-reported physical health status and mental health status were lower in AF individuals and were related to depressive symptom severity.

In our sample we demonstrated that a physician-diagnosed history of depression and current depressive symptoms were slightly more frequent in participants with AF compared to individuals without documented AF. The Somatic symptom dimension of depression accounted for most of the observed associations similar to other cardiovascular diseases [18], [12], [15]. Mostly behavioural mediators, such as reduced physical activity, unhealthy lifestyle and poor treatment adherence have been made responsible for cardiovascular disease progression and adverse prognosis in patients with depressive symptoms [19]. Disease related physical impairment may also aggravate depressive symptoms and negative health behaviours. A small study in community-dwelling elderly patients reported poorer physical health-related quality of life in AF [20]. We found a comparable magnitude of the association of self-reported impaired physical and mental health in participants with AF in our sample. To further elucidate the role of self-reported reduced physical health in AF, we examined heart failure as a potential underlying cause of physical impairment. Heart failure is a common comorbid condition in AF individuals [21], and may significantly affect physical symptom status. Depression is frequent in heart failure patients and it carries a risk factor for adverse outcome [22]. Neither somatic nor cognitive depressive symptom dimensions differed significantly depending on presence of heart failure. In contrast, physical as well as mental health status were rated lower in the subgroup of individuals with manifest heart failure. When main regression analyses were adjusted for prevalent heart failure, results remained stable indicating that heart failure did not account for depressive symptoms to a large extent despite an association with physical and mental health.

In AF patients psychological distress may drive AF-specific symptom severity and healthcare resource utilization independent of AF burden [23], [24]. To understand whether the awareness of the diagnosis of AF is related to more severe depressive symptoms we performed subgroup analyses in individuals with a self-reported history of AF. Results were comparable to the main analyses after exclusion of individuals who have not been aware of their disease. This observation renders it unlikely that depression is only an emotional consequence of labeling oneself as a cardiac case.

Whether depressive symptom dimensions in atrial fibrillation individuals are mediated through elevated inflammatory activity was assessed by CRP measurement. Inflammatory pathways underlie both conditions, AF [25] and depression [26]. Elevated inflammatory activity in depression has been made responsible for adverse outcomes in patients with manifest cardiovascular disease [27]. In a small study in AF patients, type D personality, i.e. the compound of negative affectivity and social inhibition, and CRP were related to health related quality of life [28]. In our analyses, we did not observe significant differences for associations of depressive symptom dimensions with higher CRP concentrations or after adjustment for circulating CRP. Thus it seems that correlations are not primarily driven by inflammation despite a potential common background of both disease states.

As a previous study found that elevated depression symptoms and marital status had an additive predictive value for cardiovascular and arrhythmic death [4], we analyzed whether the association of AF with depression was mediated through living in a partnership. Indeed, a history of depression and self rated poor mental health status were more prominent among persons with AF living without a current partnership. Thus, lack of social support respectively living without a partner might moderate the association of depression with AF.

Our findings are in line with recent publications. For several common diseases it could be demonstrated that concomitant depression is related to worse overall health rating than the disease alone [29]. The obvious goal of improving mental well-being in AF needs to be examined for its efficiency with respect to quality of life and healthcare costs.

We did not observe significant interactions by age or sex. Whereas age seems to play a minor role for the prevalence of depression, female sex is a strong risk factor for depressive symptoms [7], [29]. The number of women with AF in our sample, however, was comparatively small and statistical significance may not have been reached.

The current study results will have potential public health impact for AF. In health professionals, awareness of depression and mental health status in atrial fibrillation needs to increase. Systematic screening may enhance our understanding of prevalence and sequelae of depression in AF. The identification of depression is important because it impairs subjective mental and physical health status in AF. Such symptoms can be positively affected by lifestyle interventions, psychotherapy, and medication [30], [31]. Changes in depressive symptoms could lead to better quality of life, and might affect outcome in AF individuals in the general population. Importantly, proper treatment and prevention of AF recurrence has the potential to significantly reduce depressive symptoms and finally, reduce healthcare costs [32].

## Supporting Information

Table S1 Characteristics of the sample by AF status weighted for population of the geographic region.
(DOCX)

Table S2 Characteristics of participants with a diagnosis of AF stratified by awareness of atrial fibrillation.
(DOCX)

Table S3 Multivariable logistic regression of depressive symptom dimensions in relation to AF for individuals with a self-reported history of AF (N = 265).
(DOCX)

Table S4 Multivariable logistic regression of depressive symptom dimensions in relation to self-rated physical well-being (A) and mental well-being (B) for individuals with AF (N = 309).
(DOCX)
