# Supplementary material for: Depression in Atrial Fibrillation in the General Population
Source: PLoS One. 2013 Dec 4;8(12):e79109. doi: 10.1371/journal.pone.0079109 (PMC3850915; doi:10.1371/journal.pone.0079109)
Supplement: Table S1 — Characteristics of the sample by AF status weighted for population of the geographic region. (DOCX) [file pone.0079109.s001.docx]

**SUPPLEMENT MATERIAL**

**Table S2. Characteristics of participants with a diagnosis of AF stratified by awareness of atrial fibrillation.**

| Variable | Self-reported and ECG documentation of AF  N=76 | Self-reported AF,  rhythm other than AF on ECG  N=189 | Newly diagnosed  AF on ECG  N=44 |
| --- | --- | --- | --- |
| Age, years | 67.7±5.9 | 63.2±8.7 | 66.2±7.6 |
| Female gender, N (%) | 16 (21.1) | 65 (34.4) | 12 (27.3) |
| Body mass index, kg/m² | 28.45 (26.42/32.29) | 28.10 (25.26/31.68) | 31.21 (26.36/34.19) |
| Systolic blood pressure (mmHg) | 130.8±16.4 | 133.3±18.0 | 133.8±15.9 |
| Smoking, N (%) | 9 (12) | 24 (12.8) | 8 (18.2) |
| Diabetes, N (%) | 12 (15.8) | 19 (10.1) | 16 (36.4) |
| Hypertension, N (%) | 59 (77.6) | 130 (68.8) | 34 (77.3) |
| Family history of myocardial infarction, N (%) | 8 (10.5) | 38 (20.1) | 10 (22.7) |
| History of heart failure, N (%) | 14 (18.4) | 22 (11.6) | 1 (2.3) |
| History of myocardial infarction, N (%) | 12 (16.2) | 24 (13.0) | 6 (13.6) |
| CRP, mg/ L | 4.7±5.4 | 3.1±3.5 | 4.0±3.6 |
| Depressive Symptoms |  |  |  |
| History of depression, N (%) | 10 (13.3) | 33 (17.9) | 6 (14.0) |
| PHQ-9 | 3 (1/5) | 4 (2/6) | 4 (2/6) |
| Caseness of depression, PHQ-9 ≥10 | 3 (4.0) | 13 (7.1) | 2 (4.8) |
| Somatic depression (0-12) | 2 (1/3) | 2 (1/4) | 2 (1/3) |
| Cognitive depression (0-12) | 1 (0/2) | 1 (0/2) | 1 (0/3) |
| Partnership, N (%) | 60 (78.9) | 157 (83.1) | 36 (81.8) |
| Socioeconomic status | 11.7±4.6 | 12.2±4.7 | 10.0±4.2 |
| Mental health status |  |  |  |
| Very good mental health status, N (%) | 14 (18.4) | 27 (14.3) | 3 (6.8) |
| Good mental health status, N (%) | 41 (53.9) | 125 (66.1) | 33 (75) |
| Fair mental health status, N (%) | 21 (27.6) | 29 (15.3) | 8 (18.2) |
| Poor mental health status, N (%) | 0 (0.0) | 8 (4.2) | 0 (0.0) |
| Physical health status |  |  |  |
| Very good physical health status, N (%) | 4 (5.3) | 14 (7.4) | 3 (6.8) |
| Good physical health status, N (%) | 45 (59.2) | 112 (59.3) | 23 (52.3) |
| Fair physical health status, N (%) | 23 (30.3) | 48 (25.4) | 15 (34.1) |
| Poor physical health status, N (%) | 4 (5.3) | 15 (7.9) | 3 (6.8) |

The numbers are mean and standard deviation for continuous variables, or median (25^th^/75^th^ percentile) for variables with a skewed distribution. Number and percent are shown for categorical variables.

CRP stands for C-reactive protein and PHQ stands for Patient Health Questionnaire.
