# Supplementary material for: Depression in Atrial Fibrillation in the General Population
Source: PLoS One. 2013 Dec 4;8(12):e79109. doi: 10.1371/journal.pone.0079109 (PMC3850915; doi:10.1371/journal.pone.0079109)
Supplement: Table S3 — Multivariable logistic regression of depressive symptom dimensions in relation to AF for individuals with a self-reported history of AF (N = 265). (DOCX) [file pone.0079109.s003.docx]

**SUPPLEMENT MATERIAL**

**Table S3. Multivariable logistic regression of depressive symptom dimensions in relation to AF for individuals with a self-reported history of AF (N=265).**

| Variable | Model R² | Odds Ratio | *P* Value |
| --- | --- | --- | --- |
| History of depression | 0.11  0.14 | 1.44 (1.02-2.02)  1.25 (0.88-1.78) | 0.037  0.21 |
| Severity of depression (PHQ-9) | 0.12  0.15 | 1.06 (1.03-1.10)  1.04 (1.01-1.08) | <0.001  0.023 |
| Caseness of depression (PHQ-9 ≥10) | 0.11  0.14 | 1.20 (0.71-2.02)  0.97 (0.56-1.68) | 0.50  0.92 |
| Somatic depression (0-12) | 0.12  0.15 | 1.13 (1.06-1.20)  1.09 (1.02-1.16) | <0.001  0.0077 |
| Cognitive depression (0-15) | 0.12  0.15 | 1.08 (1.01-1.15)  1.05 (0.98-1.12) | 0.022  0.16 |
| Physical health status  (very good/good vs. fair/poor) | 0.12  0.15 | 0.49 (0.38-0.64)  0.54 (0.41-0.72) | <0.001  <0.001 |
| Mental health status  (very good/good vs. fair/poor) | 0.12  0.15 | 0.56 (0.41-0.76)  0.60 (0.44-0.82) | <0.001  0.0015 |

Odds ratios are across categories or for the dichotomous variable as indicated. Self-reported history atrial fibrillation was used as dependent variable. Multivariable-adjusted models included age, sex (upper row) and age, sex, body mass index, systolic blood pressure, antihypertensive medication, diabetes, current smoking and a history of myocardial infarction, dyslipidemia (lower row) and respective model R² values.
