# Supplementary material for: Depression in Atrial Fibrillation in the General Population
Source: PLoS One. 2013 Dec 4;8(12):e79109. doi: 10.1371/journal.pone.0079109 (PMC3850915; doi:10.1371/journal.pone.0079109)
Supplement: Table S4 — Multivariable logistic regression of depressive symptom dimensions in relation to self-rated physical well-being (A) and mental well-being (B) for individuals with AF (N = 309). (DOCX) [file pone.0079109.s004.docx]

**SUPPLEMENT MATERIAL**

**Table S4. Multivariable logistic regression of depressive symptom dimensions in relation to self-rated physical well-being and mental well-being for individuals with AF (N=309).**

| **Dependent variables** | **Self-rated physical well-being** (very good/good versus fair/bad) | | | **Self-rated mental well-being** (very good/good versus fair/bad) | | |
| --- | --- | --- | --- | --- | --- | --- |
|  | Model R² | Odds Ratio | *P* Value | Model R² | Odds Ratio | *P* Value |
| **Predictor variables** |  |  |  |  |  |  |
| History of depression | 0.09 | 0.40 (0.21-0.77) | 0.0057 | 0.21 | 0.15 (0.08-0.30) | <0.001 |
|  | 0.14 | 0.38 (0.19-0.76) | 0.0065 | 0.23 | 0.14 (0.07-0.30) | <0.001 |
| Severity of depression (PHQ-9) | 0.23 | 0.77 (0.70-0.84) | <0.001 | 0.35 | 0.70 (0.62-0.78) | <0.001 |
|  | 0.28 | 0.75 (0.68-0.83) | <0.001 | 0.35 | 0.69 (0.61-0.77) | <0.001 |
| Caseness of depression (PHQ-9 ≥10) | 0.12 | 0.13 (0.04-0.43) | <0.001 | 0.26 | 0.02 (0-0.10) | <0.001 |
|  | 0.15 | 0.14 (0.04-0.47) | 0.0015 | 0.26 | 0.02 (0-0.11) | <0.001 |
| Somatic depression (0-12) | 0.20 | 0.66 (0.57-0.77) | <0.001 | 0.19 | 0.70 (0.60-0.81) | <0.001 |
|  | 0.26 | 0.64 (0.54-0.75) | <0.001 | 0.21 | 0.69 (0.58-0.81) | <0.001 |
| Cognitive depression (0-15) | 0.18 | 0.70 (0.60-0.81) | <0.001 | 0.40 | 0.49 (0.40-0.60) | <0.001 |
|  | 0.22 | 0.69 (0.59-0.80) | <0.001 | 0.40 | 0.48 (0.39-0.60) | <0.001 |
| Partnership status | 0.07 | 1.38 (0.75-2.54) | 0.30 | 0.08 | 1.63 (0.83-3.19) | 0.15 |
|  | 0.12 | 1.38 (0.73-2.61) | 0.32 | 0.10 | 1.64 (0.82-3.25) | 0.16 |
| Socioeconomic status | 0.06 | 1.04 (0.98-1.09) |  | 0.06 | 1.01 (0.95-1.08) | 0.75 |
|  | 0.11 | 1.03 (0.97-1.09) | 0.38 | 0.09 | 1.02 (0.96-1.09) | 0.54 |

Multivariable-adjusted models included age, sex (upper row) and age, sex, body mass index, systolic blood pressure, antihypertensive medication, diabetes, current smoking and a history of myocardial infarction, dyslipidemia (lower row) and respective model R² values.
